# p75NTR^−/−^ mice exhibit an alveolar bone loss phenotype and inhibited PI3K/Akt/β‐catenin pathway

**DOI:** 10.1111/cpr.12800

**Published:** 2020-03-25

**Authors:** Yingying Wang, Kun Yang, Gang Li, Rui Liu, Junyu Liu, Jun Li, Mengying Tang, Manzhu Zhao, Jinlin Song, Xiujie Wen

**Affiliations:** ^1^ Department of Stomatology Daping Hospital Army Medical University (Third Military Medical University) Chongqing China; ^2^ Department of Periodontology Stomatological Hospital Zunyi Medical University Zunyi China; ^3^ College of Stomatology Chongqing Medical University Chongqing China; ^4^ Hospital of Stomatology Southwest Medical University Luzhou China

## Abstract

**Objectives:**

The aim of this study was to investigate the role of p75 neurotrophin receptor (p75NTR) in regulating the mouse alveolar bone development and the mineralization potential of murine ectomesenchymal stem cells (EMSCs). Moreover, we tried to explore the underlying mechanisms associated with the PI3K/Akt/β‐catenin pathway.

**Materials and methods:**

p75NTR knockout (p75NTR^−/−^) mice and wild‐type (WT) littermates were used. E12.5d p75NTR^−/−^ and WT EMSCs were isolated in the same pregnant p75NTR^‐/+^ mice from embryonic maxillofacial processes separately. Mouse alveolar bone mass was evaluated using micro‐CT. Differential osteogenic differentiation pathways between p75NTR^−/−^ and WT EMSCs were analysed by RNA‐sequencing. The PI3K inhibitor LY294002 and PI3K agonist 740Y‐P were used to regulate the PI3K/Akt pathway in EMSCs. p75NTR overexpression lentiviruses, p75NTR knock‐down lentiviruses and recombined mouse NGF were used to transfect cells.

**Results:**

The alveolar bone mass was found reduced in the p75NTR knockout mouse comparing to the WT mouse. During mineralization induction, p75NTR^−/−^ EMSCs displayed decreased osteogenic capacity and downregulated PI3K/Akt/β‐catenin signalling. The PI3K/Akt/β‐catenin pathway positively regulates the potential of differential mineralization in EMSCs. The promotive effect of p75NTR overexpression can be attenuated by LY294002, while the inhibitory effect of p75NTR knock‐down on Runx2 and Col1 expression can be reversed by 740Y‐P.

**Conclusion:**

Deletion of p75NTR reduced alveolar bone mass in mice. P75NTR positively regulated the osteogenic differentiation of EMSCs via enhancing the PI3K/Akt/β‐catenin pathway.

## 1 INTRODUCTION

P75 neurotrophin receptor (p75NTR), a single membrane‐spanning protein in the TNF receptor family,^1^ is a low‐affinity receptor capable of binding all neurotrophins. Previous studies have shown that p75NTR plays critical roles in the morphogenesis and development of many embryonic and adult tissues.^2^, ^3^ A series of reports have demonstrated that p75NTR can carry out a wide range of cellular functions depending on the cellular context, including cell proliferation,^4^, ^5^, ^6^ cell death^7^, ^8^, ^9^ and multi‐differentiation.^10^, ^11^, ^12^, ^13^ Additionally, increasing numbers of studies have demonstrated that p75NTR acts as a key regulator of osteogenesis in many cell lines.^11^, ^14^, ^15^, ^16^, ^17^, ^18^ Yoshikazu Mikami et al reported that p75NTR overexpression significantly enhanced osteoblast differentiation in the MC3T3‐E1 pre‐osteoblast cell line.^16^ In contrast, p75NTR inhibited the osteogenic differentiation of C3H10T cells.^17^ These results demonstrate the functional diversity of p75NTR, which regulates the mineralization capacity of diverse cell lines.

The cranial neural crest (CNC), a population of transient migratory cells originated from the dorsal aspect of the neural tube during embryonic development, contributes significantly to the formation of craniofacial structures.^19^ At the early stages of embryogenesis, CNC cells first migrate into the first branchial arch and thereafter reside within the maxillary and mandibular prominences, where they form ectomesenchymal stem cells (EMSCs). Therefore, EMSCs are the progenitor cells of craniofacial hard tissues including the maxilla, mandible and all the tooth tissues except enamel.^20^

It has been reported that p75NTR might participate in the regulation of rat tooth morphogenesis, especially dental hard tissue formation.^21^ Xing et al^22^ found that p75NTR‐positive EMSCs showed greater odontogenic differentiation ability than p75NTR‐negative EMSCs both in vivo and in vitro. They further demonstrated that a high expression level of Smad4 in p75NTR‐positve EMSCs was responsible for this increased odontogenic differentiation potential. Moreover, our previous study demonstrated that p75NTR and its binding to Mage‐D1 play crucial roles in the mineralization and differentiation of rat EMSCs in the initial tooth development stage.^23^ Therefore, we hypothesized that p75NTR is also involved in development of the mandible, which originates from EMSCs as well.

PI3K/Akt signalling is involved in numerous cellular processes in multiple cell types. Following PI3K activation, phosphatidylinositol 3, 4, 5 triphosphate (PIP3), the lipid product of PI3K, recruits Akt to the plasma membrane where Akt is phosphorylated and activated.^24^, ^25^, ^26^ The PI3K/Akt pathway has previously been found to play important roles in osteoblast differentiation and bone tissue metabolism.^27^, ^28^, ^29^ In addition, β‐catenin signalling is involved in bone formation. Reports have shown that the inhibitory phosphorylation of GSK‐3β caused by PI3K/Akt signalling activates β‐catenin, which further increases the expression of Runx2.^30^ However, whether PI3K/Akt/β‐catenin pathway is required for the osteogenic differentiation of EMSCs remains unknown.

In this study, we investigated whether p75NTR regulates the alveolar bone development and osteogenic differentiation of murine EMSCs and further explored the underlying mechanisms associated with the PI3K/Akt/β‐catenin pathway.

## 2 MATERIALS AND METHODS

### 2.1 Animals and tissue preparation

p75NTR knockout (p75NTR^−/−^) mice used in this study were obtained from The Jackson Laboratory. These mutant mice that exhibit the targeted deletion of exon III of the p75NTR locus does not express functional full‐length p75NTR.^31^ All mice were housed under standard conditions in the Third Military Medical University Animal Laboratory. All experiments were performed in accordance with protocols approved by the Medical Ethics Committee of the Third Military Medical University. Every 3 pairs of male wild‐type and p75NTR^−/−^ mice were used for extraction of mandibular RNA and protein, and individual mouse sample were used for experiment.

### 2.2 Microscopic computed tomography (micro‐CT) analysis and serum biochemistry

All mandibles of the 1‐month‐old (n = 5) and 4‐month‐old (n = 5) male wild‐type (WT) mice and their male p75NTR^−/−^ littermates were dissected free of soft tissue and scanned by micro‐CT (viva CT 40, Scanco Medical AG; Switzerland). Three‐dimensional (3D) microstructural parameters were calculated using MicroView software version 2.2. Alveolar bone microstructural parameters were measured directly from the region of interest (ROI) which was manually established in the inter‐radicular septa of the mandibular first molar (M1). The sampling region started coronally from the M1 inter‐radicular septa to root apex direction after 50 continuous scan slices, and 50 continuous scan slices with the same shape and tissue volume were considered as the ROI in each specimen. Three‐dimensional micro‐architecture parameters of the ROI were also evaluated.

Serum calcium and phosphorus were measured with a Calcium Assay Kit (Nanjing Jiancheng Bioengineering Institute, China) and a Phosphate Assay Kit (Nanjing Jiancheng Bioengineering Institute, China), respectively. Serum parathyroid hormone (PTH) and calcitonin (CT) were detected using a PTH ELISA Kit (RayBiotech, USA) and a Calcitonin Assay Kit (Cloud‐Clone Corp. China), respectively.

### 2.3 Extraction and identification of E12.5d WT and p75NTR^−/−^ EMSCs

p75NTR heterozygous pregnant mice at E12.5d were obtained, and the embryonic maxillofacial processes were dissected from each embryo separately, while the remaining tissue was used for genotyping as previously reported.^31^ Then, primary EMSCs were extracted according to our previous study.^23^ Briefly, we used 1% trypsin/1 mmol/L EDTA solution (Sigma, USA) to digest the minced tissue at 37°C for 10 minutes and then stopped digestion with DMEM/F12 (Gibco, USA) containing 10% FBS (Gibco, USA). The cell suspension was filtered to remove tissue debris using a 75um mesh filter. Then, cell resuspended in above culture medium supplemented with 1% antibiotics (100 ug/ml penicillin and 100 µg/mL streptomycin). Cells were cultured at 37°C in a 5% CO_2_ humidified incubator.

Flow cytometry analysis was used to detect the cell surface markers on E12.5d WT and p75NTR^−/−^ EMSCs as described previously.^32^ The primary antibodies including anti‐mouse CD14, anti‐mouse CD90, anti‐mouse CD146, anti‐mouse CD166, anti‐mouse CD45 (1:100; Santa Cruz, USA) and anti‐mouse p75NTR‐FITC (1:100; Abcam, UK) were used to identify EMSCs.

### 2.4 CCK‐8 proliferation and colony formation assay

Cell Counting Kit‐8 (CCK‐8; Dojindo Kagaku Co., Japan) was used to analyse proliferation rate of E12.5d WT and p75NTR^−/−^ EMSCs, according to the manufacturer's protocols. Briefly, E12.5d WT and p75NTR^−/−^ EMSCs were seeded at 2 × 10^3^ cells/well in 96‐well plates and the culture medium was replaced daily. CCK‐8 solution was added into each well and further cultured for 2 hours at 37°C. Absorbance was measured using a microplate reader at 450 nm to determine the number of viable cells in each well. Absorbance was detected for 7 days, and cell proliferation was represented as mean ± SD of absorbance for five wells from each group. The E12.5d WT and p75NTR^−/−^ EMSCs were seeded at 150 cells/well into 6‐well plates and cultured for 14 days. Then, the cells were stained with Crystal Violet Staining Kit (Beyotime) according to the manufacturer's protocol, and the number of colonies was counted.

### 2.5 Culture and osteogenic induction of E12.5d WT and p75NTR^−/−^ EMSCs

E12.5d WT and p75NTR^−/−^ EMSCs were cultured in DMEM/F12 containing 10% FBS and 1% antibiotics. Third passage WT and p75NTR^−/−^ EMSCs were used for the experiments. The osteogenic differentiation medium (α‐MEM supplemented with 10% FBS, 1% antibiotics, 10 mmol/L β‐glycerol phosphate, 100 nmol/L dexamethasone, and 50 mg/mL ascorbic acid) was replaced every 3 days. To regulate the PI3K/Akt pathway, the PI3K inhibitor LY294002 (10 mmol/L, MCE, USA) and PI3K agonist 740Y‐P (30 µg/mL, Selleck Chemicals, USA) were added to the medium. To detect the role of NGF in osteogenic differentiation of EMSC, exogenous NGF (100 ng/mL, R&D systems, USA) were added to the osteogenic differentiation medium.

### 2.6 Plasmid constructs and lentivirus transfection

Coding region of p75NTR was amplified from cDNAs of murine EMSCs and then was cloned into vector pLJM1 (Addgene #19319). The plasmid pLJM1‐p75NTR was co‐transfected with lentivirus helper plasmids (psPAX2 and pCMV‐VSV‐G) into HEK‐293T cells using Lipofectamine 2000 (Invitrogen, USA) according to the manufacturer's protocol. Supernatants containing virus were collected 60 hours after transfection and then infected EMSCs in the presence of 8 μg/mL polybrene (Solarbio, China). The cells were selected with 2 μg/mL puromycin 24 hours later. To knock down p75NTR expression, the plasmid was established with pLKO.1 (Addgene #8453) according to the manufacturer's protocol. The shRNA sequence is 5'‐ AAGGAGACATGTTCCACAGGC‐3'. Packaging of these knock‐down lentiviruses is the same as that of expression lentiviruses. All plasmids were sequenced.

### 2.7 ELISA of NGF

NGF was quantified in the osteogenic induction medium supernatant of WT EMSCs. A commercially available NGF‐specific, highly‐sensitive ELISA kit (Mlbio, China) was used following the manufacturer's instructions. Absorbance was measured using a microplate reader at 450 nm. The detection range was 12.5‐400 pg/mL.

### 2.8 Alkaline phosphatase and Alizarin Red staining

EMSCs were cultured with the osteogenic induction medium. On days 7 and 21, the cells were stained using an alkaline phosphatase (ALP) staining kit (Beyotime, China) or Alizarin Red (Sangon, China) separately, according to the previous study.^23^

### 2.9 RNA–sequencing (RNA‐Seq)

RNA‐Seq was performed by Novogene Co.,Ltd. (Beijing, China). Briefly, total RNA was extracted from 3 pairs of WT and p75NTR^−/−^ EMSCs after 7 days of osteogenic induction. Three micrograms of RNA per sample was used for RNA sample preparations. An NEBNext UltraTM RNA Library Prep Kit for Illumina (New England Biolabs, USA) was used to generate sequencing libraries, and index codes were added to attribute sequences to each sample. The library quality was assessed with an Agilent Bioanalyzer 2100 system. Clustering of the index‐coded samples was performed on a cBot Cluster Generation System using a TruSeq PE Cluster Kit v3‐cBot‐HS (Illumina). The Illumina HiSeq platform was used to sequence the library preparations and 125 bp/150 bp paired‐end reads were generated. Then, clean, high‐quality data were analysed. HISAT2 was used as the mapping tool. We used the expected number of fragments per kilobase of transcript sequence per million base pairs sequenced (FPKM) to estimate gene expression levels. Analysis of differential expression was performed using the DESeq2 R package. Benjamini and Hochberg's approach was used to adjust P‐values to control the false discovery rate. An adjusted *P*‐value < .05 indicated statistical significance. Genomes (KEGG) analysis was applied with the clusterProfiler R package, and differentially expressed genes with corrected *P*‐values < .05 were considered significantly enriched. Gene networks were generated based on gene connectivity and aligned against the KEGG database (http://www.genome.jp/kegg/).

### 2.10 RT‐PCR

RT‐PCR was performed as previously described.^33^ Sequences of the primers used are listed in Table S1.

### 2.11 Western blot and immunofluorescence assays

Western blot and immunofluorescence assays were conducted as previously described.^34^ The antibodies used in Western blot were anti‐GAPDH antibody (1:2000; Immunoway, USA), anti‐Runx2 antibody (1:1000; Abcam, USA), anti‐Collagen1(Col1) antibody (1:2000; Abcam, USA), anti‐β‐catenin antibody(1:1000; CST, USA), anti‐active β‐catenin antibody (1:1000; CST, USA), anti‐phospho‐PI3K antibody (Tyr607, 1:1000; Affinity, USA), anti‐phospho‐Akt antibody (Ser473, 1:1000; Affinity, USA), anti‐PI3K antibody (1:1000; Affinity, USA), anti‐Akt antibody (1:1000; Affinity, USA). The antibodies used in immunofluorescence were anti‐p75NTR (1:200; Abcam, USA) and goat anti‐rabbit IgG‐TRITC secondary antibody (Beyotime, China). The Western blot results were further analysed using BIO‐RAD ChenmiDoc™ XRS + or C‐DiGit^TM^ Blot Scanner from LI‐COR Biosciences. The grayscale analysis was performed with Quantity One software (Bio‐Rad; Hercules, CA, USA).

### 2.12 Statistical analysis

Data are expressed as the mean ± standard deviation. Data were analysed by two‐tailed Student's *t* test. All experiments were repeated in triplicate, and *P *< .05 was indicated statistical significance.

## 3 RESULTS

### 3.1 p75NTR^−/−^ mice exhibit an alveolar bone loss phenotype

**FIGURE 1:**
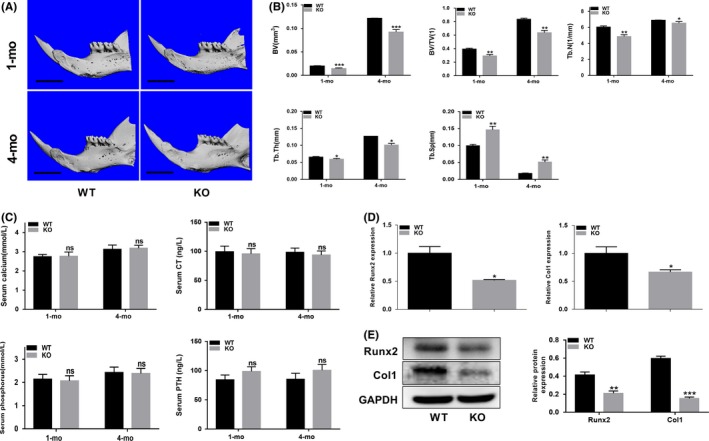
p75NTR^−/−^ mice exhibit an alveolar bone loss phenotype. (A) Micro‐CT three‐dimensional images of alveolar bone in 1‐month‐old and 4‐month‐old male wild‐type (WT) and p75NTR^−/−^ mice. Scale bar represents 2.0 mm. (B) Bone histomorphometry of the alveolar bone. Bone volume (BV), bone volume/total volume (BV/TV), trabecular number (Tb.N), trabecular thickness (Tb.Th) and trabecular separation (Tb.Sp) are shown. (C) Serum levels of calcium, phosphorus, parathyroid hormone (PTH) and calcitonin (CT) in 1‐month‐old and 4‐month‐old WT and p75NTR^−/−^ mice. (D) mRNA and (E) protein levels of Runx2 and Col1 in 4‐month‐old WT and p75NTR^−/−^ mice alveolar bone were detected by real‐time PCR and Western blot analysis, respectively, GAPDH were used as the reference gene. Grayscale analysis was performed, and the levels of the indicated proteins are expressed relative to the levels of GAPDH. KO represent p75NTR^−/−^. Data are shown as mean ± SD from three independent experiments. *P < .05, **P < .01, ***P < .001, ns = no significant difference

To determine whether p75NTR plays a role in alveolar bone development, we first tested the bone mass in 1‐month‐old and 4‐month‐old male WT mice and their male p75NTR^−/−^ littermates (Figure 1A). Micro‐CT scanning was used to visualize the ROI in alveolar bone samples from these two kinds of mice. Bone mass in the p75NTR^−/−^ mice was strikingly reduced, with significantly decreased bone volume (BV) and BV/total volume (TV) (Figure 1B) comparing to the bone mass in the wild‐type control mice. P75NTR^−/−^ mice also exhibited deteriorated bone microarchitecture, with a significant reduction in trabecular number (Tb.N) and trabecular thickness (Tb.Th) (Figure 1B). Furthermore, the knockout mice showed increased trabecular separation (Tb.Sp) (Figure 1B) that further demonstrated their bone loss phenotype. We further detected the expression of Runx2 and Col1 in 4‐month‐old mice by extracting total mandibular bone RNA and proteins, finding that both Runx2 and Col1 levels were lower in p75NTR^−/−^ mice (Figure 1D and E), indicating that deletion of p75NTR inhibits osteogenic differentiation in mandibular bone.

To determine the mechanisms leading to the decreased bone mass observed in p75NTR^−/−^ mice, we examined serum calcium, phosphorus, PTH, and CT levels in the two kinds of mice. No significant differences in the levels of these parameters were detected (Figure 1C), implying that the bone loss observed in p75NTR^−/−^ mice is not associated with calcium/phosphate homeostasis.

### 3.2 The comparison of osteogenic differentiation of WT and p75NTR^−/−^ EMSCs

**FIGURE 2:**
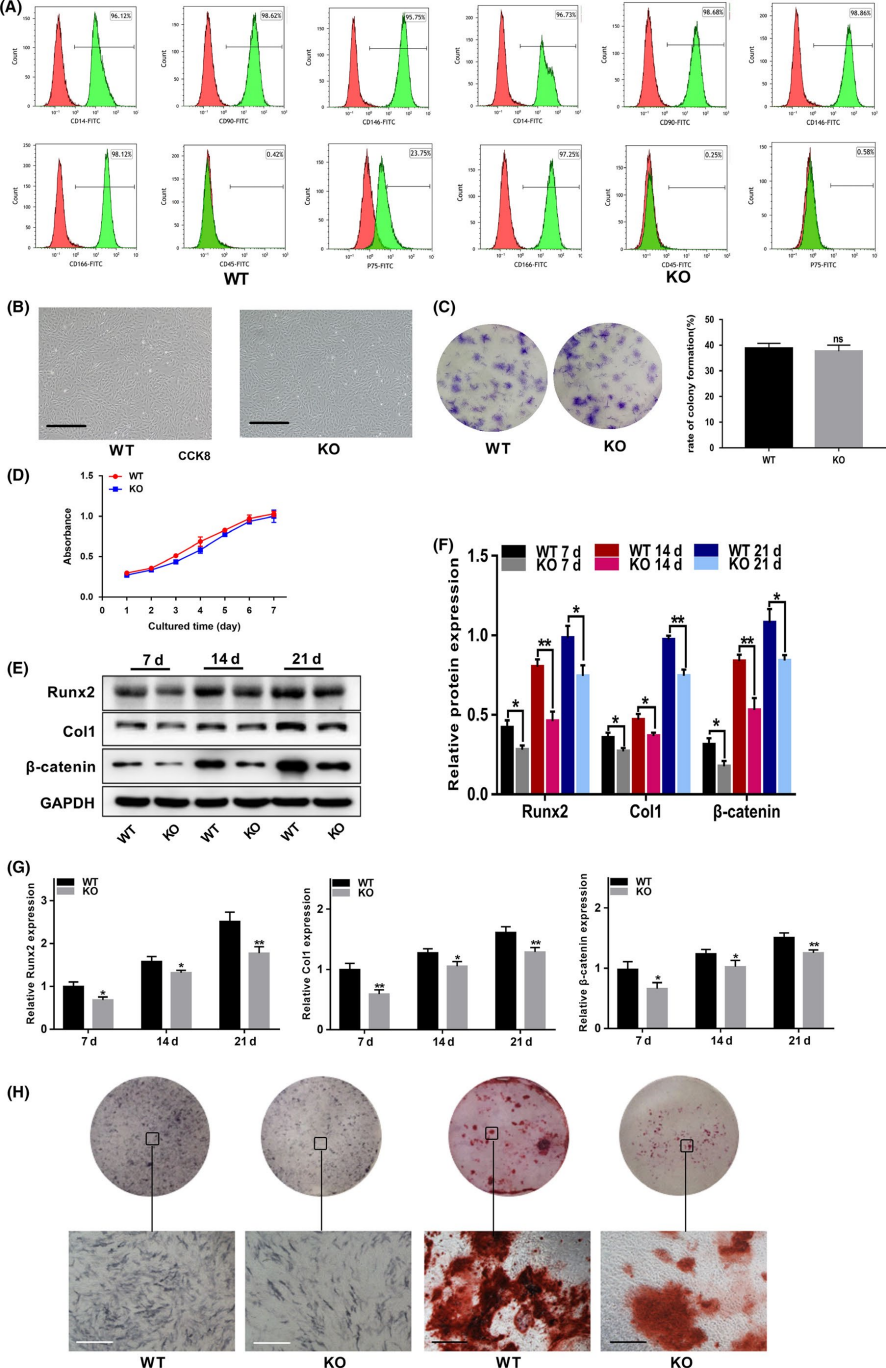
P75NTR^−/−^ EMSCs exhibit decreased osteogenic differentiation capacity compared to WT EMSCs. (A) The p75NTR and mesenchymal stem cell surface markers (CD14, CD90, CD146 and CD166) and hematopoietic markers (CD45) were detected on WT and p75NTR^−/−^ EMSCs by the Flow cytometry. (B) The third passage (P3) cells of E12.5d WT and p75NTR^−/−^ EMSCs. Scale bar represents 50 μm. (C) Representative images of colonies formed by E12.5d WT and p75NTR^−/−^ EMSCs and the analysis of colony formation. (D) The proliferation ratio of E12.5d WT and p75NTR^−/−^ EMSCs was assessed by CCK‐8. WT and p75NTR^−/−^ EMSCs were induced with osteogenic induction medium. On days 7, 14 and 21, the (E) protein levels of Runx2, Col1 and β‐catenin were detected by Western blot and (F) grayscale analysis was performed and the levels of the indicated proteins are expressed relative to the levels of GAPDH. (G) mRNA levels of Runx2, Col1 and β‐catenin were detected by real‐time PCR, respectively, using GAPDH as control. (H) On day 7, ALP staining was used to detect their potential of differential mineralization. On day 21, Alizarin Red staining was used to detect their mineralized nodules. Scale bar represents 50 μm. KO represent p75NTR^−/−^. Data are shown as mean ± SD from three independent experiments. *P < .05, **P < .01

Both WT and p75NTR^−/−^ EMSCs exhibited a fibroblast‐like morphology (Figure 2B). The mesenchymal stem cell surface markers CD14, CD90, CD146 and CD166 were all expressed in both WT and p75NTR^−/−^ EMSCs, while the haematopoietic marker CD45 was not expressed (Figure 2A). These results indicated that the WT and p75NTR^−/−^ EMSCs used in this study were mesenchymal stem cells (MSCs). Furthermore, the p75NTR expression rate in wild‐type E12.5d EMSCs was 23.75% (Figure 2A). Additionally, no significant differences of the colony formation rate (Figure 2C) and cell proliferation capacity (Figure 2D) were found between WT and p75NTR^−/−^ EMSCs.

To confirm the differences in the osteogenic differentiation abilities of WT and p75NTR^−/−^ EMSCs, the cells were induced with osteogenic induction medium. The protein and mRNA levels of Runx2 and Col1 in p75NTR^−/−^ EMSCs were much lower than those in WT EMSCs following 7, 14 and 21 days of osteogenic induction (Figure 2E, F and G). Furthermore, less ALP staining was observed in p75NTR^−/−^ EMSCs than in WT EMSCs (Figure 2H), and p75NTR^−/−^ EMSCs exhibited fewer and lighter mineralized nodules (Figure 2H). These results revealed that p75NTR^−/−^ EMSCs have a lower osteogenic differentiation capacity than WT EMSCs. During osteogenic differentiation, β‐catenin was found increasing expressed in wild‐type murine EMSCs, and the expression of β‐catenin was obviously reduced in p75NTR‐deficient EMSCs (Figure 2E, F and G).

### 3.3 The PI3K/Akt pathway is downregulated in p75NTR^−/−^ EMSCs

**FIGURE 3:**
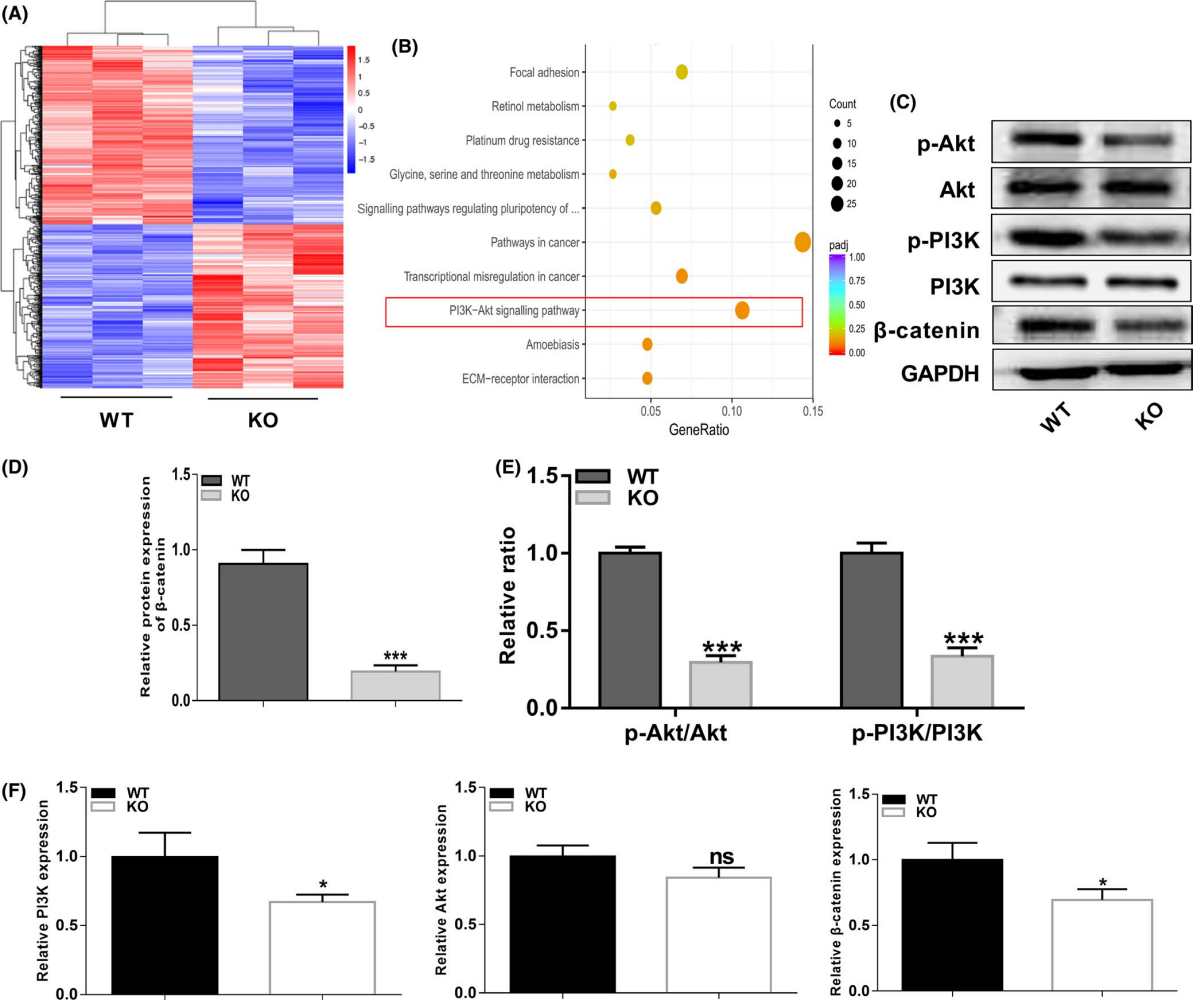
PI3K/Akt pathway is downregulated in p75NTR^−/−^ EMSCs. (A) Cluster analysis showed the differentially expressed genes between WT and p75NTR^−/−^ EMSCs after cultured in osteogenic induction medium for 7 d. (B) Kyoto Encyclopaedia of Genes and Genomes (KEGG) pathway analysis of RNA‐seq data. The top ten enriched pathways are shown. (C) The expression levels of PI3K, p‐PI3K, Akt, p‐Akt and β‐catenin were detected by Western blot analysis during osteogenic induction. Grayscale analysis was performed, and (D) the levels of β‐catenin proteins were expressed relative to the levels of GAPDH, (E) phosphorylation of PI3K and Akt were analysed, and the results were represented as fraction of the control. (F) The expression levels of PI3K, Akt and β‐catenin were detected by real‐time PCR normalized to GAPDH. All experiments were repeated at least three times. adjusted *P*‐value (Padj) < .05, *P < .05, ***P < .001, ns = no significant difference

To investigate the mechanism of EMSC differentiation, we employed RNA‐Seq to analyse the differences in gene expression between the two kinds of EMSCs during osteogenic differentiation. A total of 877 genes were differentially expressed, as shown by the cluster analysis (Figure 3A). KEGG pathway analysis showed that the differentially expressed genes were highly involved in the PI3K/Akt signalling pathway (Figure 3B). Therefore, we detected the PI3K, Akt and β‐catenin levels by real‐time PCR and Western blot analysis. Both PI3K and β‐catenin mRNA levels were significantly decreased in p75NTR^−/−^ EMSCs, while Akt mRNA expression level was not obviously decreased (Figure 3F). However, no significant differences of total PI3K and Akt protein levels were found between WT and p75NTR^−/−^ EMSCs (Figure 3C). Interestingly, protein expression of p‐PI3K, p‐Akt and β‐catenin was significantly decreased in induced p75NTR^−/−^ EMSCs (Figure 3C, D and E).

### 3.4 The PI3K/Akt/β‐catenin pathway positively regulates osteogenic differentiation in EMSCs

**FIGURE 4:**
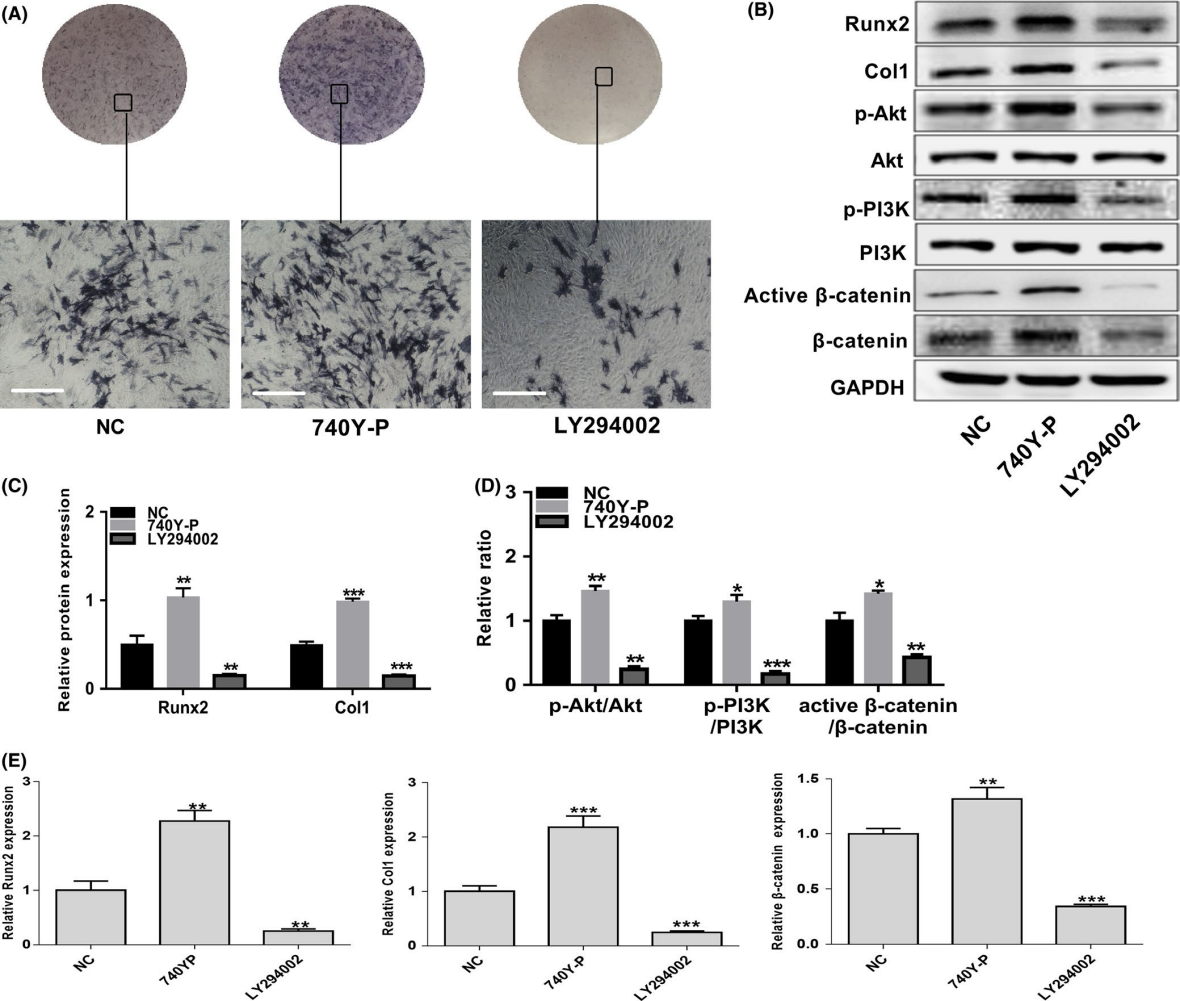
The PI3K/Akt/β‐catenin pathway positively regulates osteogenic differentiation in EMSCs. Wild‐type (NC) EMSCs were treated with the PI3K/Akt signalling PI3K agonist 740Y‐P and inhibitor LY294002 during cultured in osteogenic induction medium for 7 d. (A) ALP staining intensity was observed by optical microscopy. Scale bar represents 50 μm. (B) The protein levels of Runx2, Col1, PI3K, p‐PI3K, Akt, p‐Akt, β‐catenin and active β‐catenin were detected by Western blot analysis. Grayscale analysis was performed, and (C) the levels of Runx2 and Col1 proteins were expressed relative to the levels of GAPDH. (D) Phosphorylation of PI3K and Akt and activation of β‐catenin were analysed, and the results were represented as fraction of the control. (E) The mRNA levels of Runx2, Col1 and β‐catenin were examined by real‐time PCR normalized to GAPDH. Data are shown as mean ± SD from three independent experiments. **P < .01, **P < .01, ***P < .001

To further evaluate whether the PI3K/Akt pathway is involved in regulating EMSC osteogenic differentiation, the wild‐type EMSCs were treated with the PI3K inhibitor LY294002 and PI3K agonist 740Y‐P during osteogenic induction. The inhibitor LY294002 (10 mmol/L) markedly suppressed PI3K and Akt phosphorylation without influencing total PI3K and Akt expression, and impaired Runx2 and Col1 expression in EMSCs (Figure 4B‐E). Furthermore, the ALP staining intensity was decreased in the LY294002‐treated group (Figure 4A). In contrast, 740Y‐P (30 µg/ml) significantly activated PI3K and Akt phosphorylation without influencing total PI3K and Akt expression in EMSCs during osteogenic induction (Figure 4B, D and E). Higher protein and mRNA levels of Runx2 and Col1 were detected in 740Y‐P‐treated EMSCs (Figure 4B, C and E). Meanwhile, the ALP staining intensity was also increased in the 740Y‐P group compared with the control group (Figure 4A).

During EMSC osteogenic differentiation, β‐catenin levels were found increasingly upregulated (Figure 2E, F and G), indicating β‐catenin is involved in EMSC osteogenic differentiation. Interestingly, when PI3K/Akt pathway was regulated with agonist or inhibitor in EMSCs during osteogenic differentiation, both β‐catenin and active β‐catenin showed the same up‐ or downregulated trend (Figure 4B and D). Collectively, activation of β‐catenin was found involved in the PI3K/Akt pathway regulated osteogenic differentiation in murine EMSCs.

### 3.5 P75NTR positively regulates osteogenic differentiation of EMSCs by enhancing PI3K/Akt/β‐catenin pathway

**FIGURE 5:**
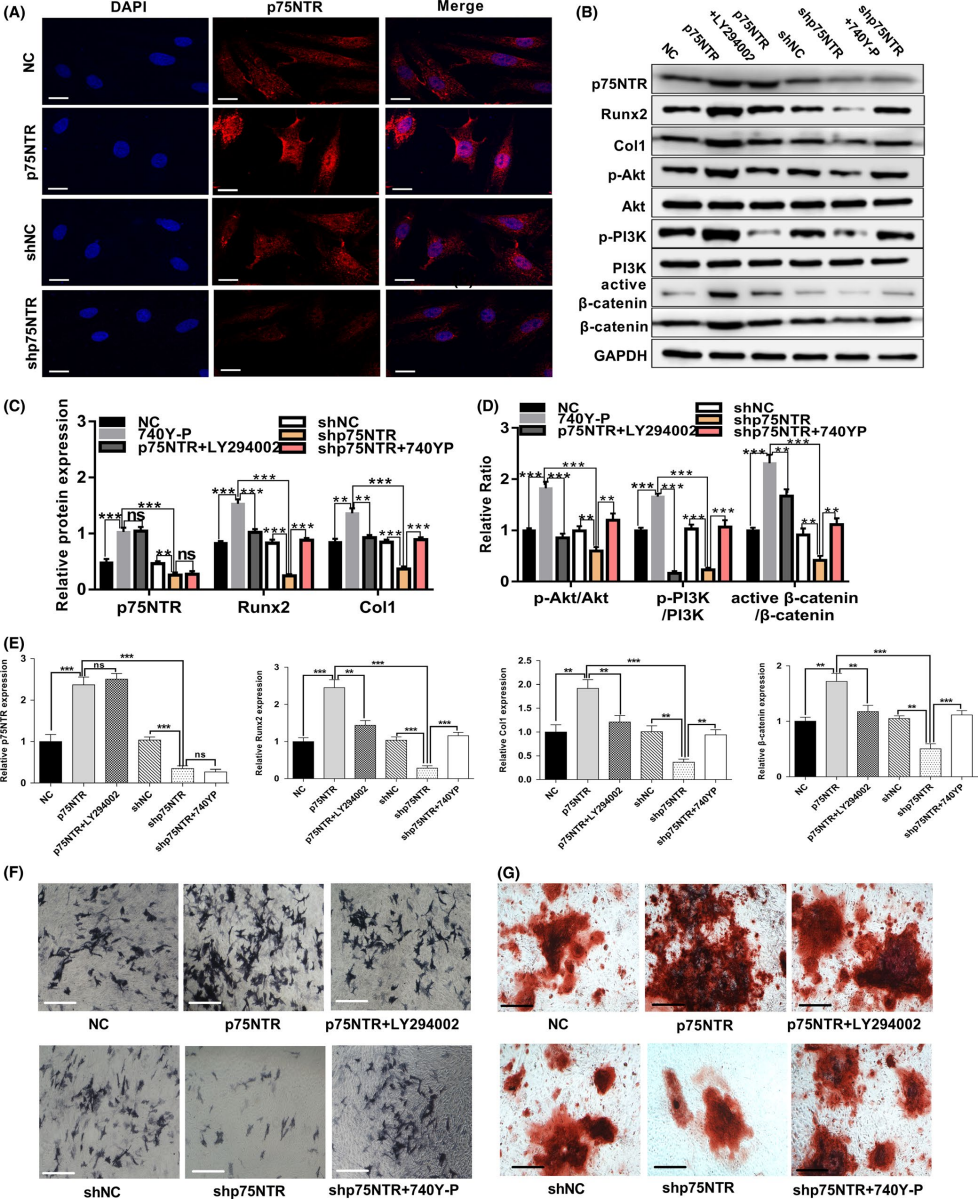
Regulation of p75NTR influenced osteogenic differentiation of EMSCs by regulating PI3K/Akt/β‐catenin pathway. (A) Immunofluorescence staining of p75NTR in p75NTR‐overexpressed (p75NTR) EMSCs and negative control EMSCs (NC), p75NTR‐knock‐down (shp75NTR) EMSCs and negative control EMSCs (shNC), Scale bar represents 25 μm. NC group EMSCs, p75NTR group EMSCs, shNC group EMSCs, shp75NTR group EMSCs, p75NTR‐overexpressed EMSCs that treated with the PI3K inhibitor LY294002 (p75NTR + LY294002) and p75NTR‐knockdown EMSCs that treated with the PI3K agonist 740Y‐P (shp75NTR + 740Y‐P) were all cultured in osteogenic induction medium. Under induction with osteogenic induction medium for 7 d, (B) the protein levels of p75NTR, Runx2, Col1, PI3K, p‐PI3K, Akt, p‐Akt, β‐catenin and active β‐catenin were detected by Western blot analysis, GAPDH used as the reference gene. Grayscale analysis was performed, and (C) the levels of P75NTR, Runx2 and Col1 proteins were expressed relative to the levels of GAPDH. (D) Phosphorylation of PI3K and Akt and activation of β‐catenin were analysed, and the results were represented as fraction of the control. (E) The mRNA levels of p75NTR, Runx2, Col1 and β‐catenin were examined by real‐time PCR normalized to GAPDH, (F) ALP staining intensity was observed by optical microscopy. Scale bar represents 50 μm. The mRNA levels of p75NTR, Runx2, Col1 and β‐catenin were examined by real‐time PCR normalized to GAPDH. (G) Under induction with osteogenic induction medium for 21 d, Alizarin Red staining was used to detect their mineralized nodules. Scale bar represents 50 μm. Data are shown as mean ± SD from three independent experiments. **P < .01, ***P < .001, ns = no significant difference

Since the PI3K/Akt/β‐catenin pathway was downregulated in p75NTR deficiency murine EMSCs and the PI3K/Akt/β‐catenin pathway positively regulates osteogenic differentiation in EMSCs, we further explored whether p75NTR could regulate the osteogenic differentiation of EMSCs through PI3K/Akt/β‐catenin pathway. We forced overexpression of p75NTR in p75NTR EMSCs group and knocked down p75NTR in shp75NTR EMSCs group. Compared with the control group, immunofluorescence assays showed that the p75NTR expression was significantly upregulated in p75NTR group and downregulated in shp75NTR group (Figure 5A). Then, the p75NTR expression levels were confirmed by Western blot and RT‐PCR assay (Figure 5B, C and E). Furthermore, we found the protein and mRNA expression levels of Runx2 and Col1 were markedly upregulated in p75NTR‐overexpressed EMSCs during osteogenic differentiation (Figure 5B, C and E). On contrary, when transfected with p75NTRshRNA, the potential of osteogenic differentiation of EMSCs was obviously downregulated (Figure 5B, C and E). These results further demonstrated that p75NTR positively regulated osteogenic differentiation ability of murine EMSCs. Then, we detected whether p75NTR could regulate the PI3K/Akt/β‐catenin pathway during osteogenic differentiation in EMSCs. While protein levels of total PI3K and Akt showed no fluctuation after p75NTR was regulated in EMSCs, p‐PI3K, p‐Akt, β‐catenin and active β‐catenin levels were obviously increased in p75NTR stably overexpressed EMSCs and markedly decreased in p75NTR knock‐down EMSCs during osteogenic differentiation (Figure 5B, D and E). Furthermore, LY294002 attenuated the increased expression levels of Runx2 and Col1 caused by p75NTR overexpression (Figure 5B, C and E). Compared with shp75NTR group, 740Y‐P markedly increased expression levels of Runx2 and Col1 in p75NTR knock‐down EMSCs during osteogenic differentiation (Figure 5B, C and E). The ALP and Alizarin Red staining were significantly increased/decreased consisting to the Western blot and RT‐PCR results (Figure 5F and G). In addition, the protein and mRNA levels of p75NTR were not regulated by LY294002 in p75NTR overexpressed EMSCs, nor by 740Y‐P in p75NTR knocked down EMSCs (Figure 5B, C and E). These results revealed that p75NTR could positively regulate osteogenic differentiation of EMSCs *via* enhancing PI3K/Akt/β‐catenin pathway.

### 3.6 Deletion of the extracellular domain of p75NTR (p75NTR^ECD^) blocked the pro‐osteogenic effects of NGF in EMSCs

**FIGURE 6:**
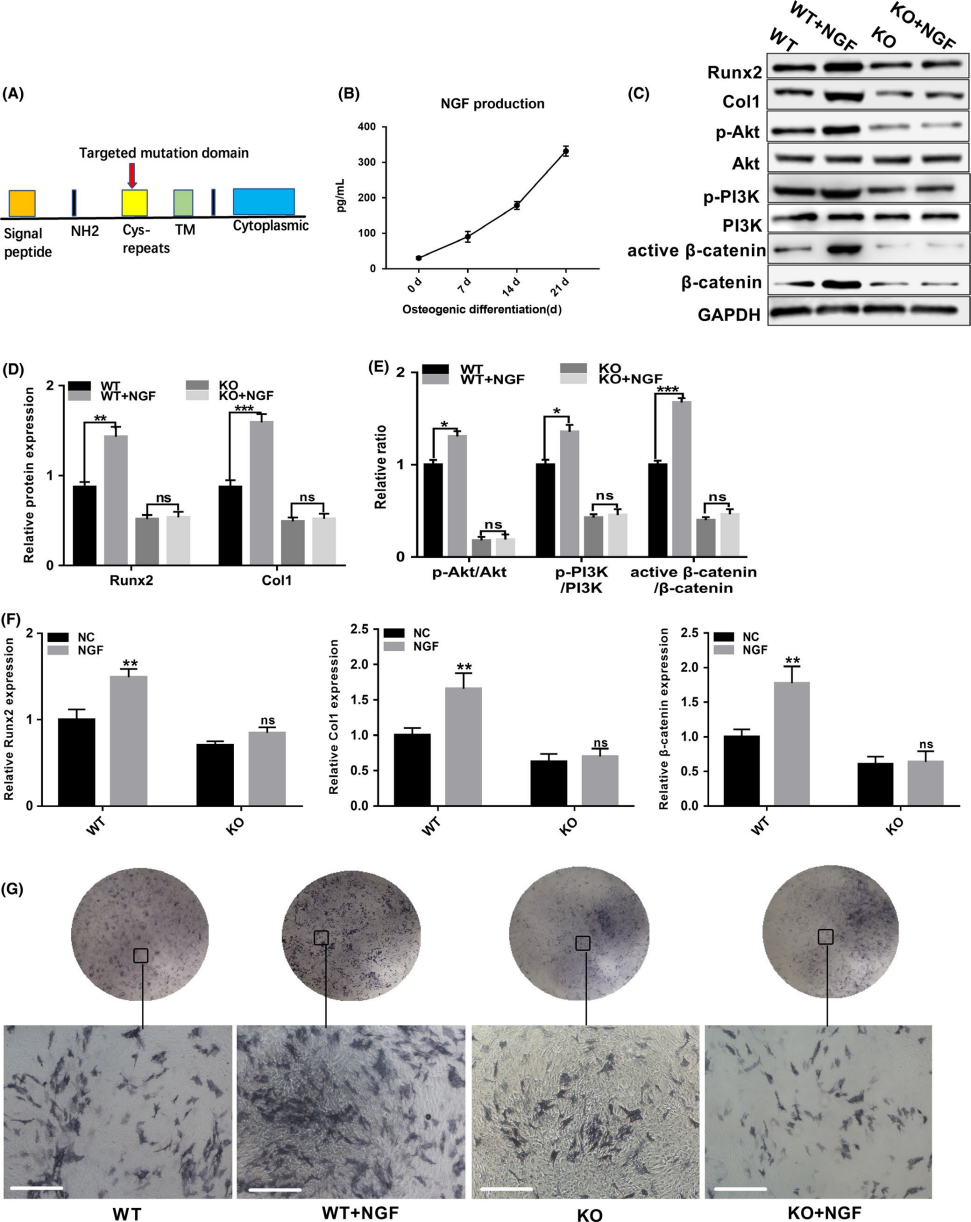
Deletion of p75NTR blocked the pro‐osteogenic effects of NGF in EMSCs. Blocked the pro‐osteogenic effects of (A) Genomic organization of the gene encoding p75NTR and the corresponding protein domains. Closed bars represent the exons. NH2, N‐terminal; Cys, cysteine; TM, transmembrane domain. The cys‐repeat domains are the binding site for NGF. (B) Supernatants were collected at osteogenic induction time points, and NGF was quantified by ELISA. Under induction with osteogenic induction medium containing exogenous NGF for 7 d, (C) the protein levels of Runx2, Col1, PI3K, p‐PI3K, Akt, p‐Akt, β‐catenin and active β‐catenin were detected by Western blot analysis, GAPDH used as the reference gene. Grayscale analysis was performed, and (D) the levels of Runx2 and Col1 proteins were expressed relative to the levels of GAPDH. (E) Phosphorylation of PI3K and Akt and activation of β‐catenin were analysed, and the results were represented as fraction of the control. (F) The mRNA levels of Runx2, Col1 and β‐catenin were examined by real‐time PCR normalized to GAPDH. (G) ALP staining intensity was observed by optical microscopy. Scale bar represents 50 μm. KO represent p75NTR^−/−^. Data are shown as mean ± SD from three independent experiments. **P < .01, **P < .01, ***P < .001 ns = no significant difference

P75NTR is a Type I transmembrane receptor that binds to neurotrophins with low affinity. The extracellular domain that contains four cysteine‐rich domains (CRDs), which are encoded by exons 2 and 3,^31^ participate in ligand binding. The mutant mice used in our study, lacking a functional p75NTR^ECD^ binding to NGF, were disrupted in the third exon encoding cysteine‐rich repeats 2, 3 and 4 (Figure 6A). ELISA assay was used to detect the endogenous NGF generated during osteogenic differentiation in EMSCs, showing an increasing production of NGF (Figure 6B). Then, we treated WT and p75NTR^−/−^ EMSCs with 100 ng/mL exogenous NGF to investigate whether the NGF could influence the potential of osteogenic differentiation in murine EMSCs. The ALP staining depth of the NGF‐treated WT group was the darker than the untreated group (Figure 6G). However, the ALP staining depth in p75NTR^−/−^ group was lighter than that in WT group, and the NGF treatment did not obviously enhance the staining depth of the p75NTR^−/−^ group (Figure 6G). The mineralization‐related genes Runx2, Col1 and the PI3K/AKT/β‐catenin‐related genes p‐PI3K, p‐Akt, β‐catenin and active β‐catenin showed the same trends assessed by Western blot and RT‐PCR (Figure 6C‐F). Additionally, neither total PI3K nor Akt expression levels were found significantly different among four groups (Figure 6C). These data suggest that the deletion of p75NTR^ECD^ blocked the pro‐osteogenic effects of NGF in osteogenic differentiation of EMSCs, which may be correlated to PI3K/AKT/β‐catenin pathway.

## 4 DISCUSSION

P75NTR was reported to play critical roles in the morphogenesis and development of many embryonic and adult tissues.^2^, ^3^ Our previous study demonstrated p75NTR could be used as a cell surface marker to sort pure CNC originated EMSCs,^10^ the progenitor cells of maxillofacial mineralized tissues and all the tooth tissues except enamel.^20^ Further studies showed that p75NTR might mediate epithelial‐mesenchymal interaction at initial tooth developmental stage^23^ and p75NTR‐positive EMSCs had a more active odontogenic differentiation ability than that of p75NTR‐negative EMSCs.^22^ These studies encouraged us to explore whether p75NTR is also involved in the development of mandible, which originates from EMSCs as well. However, the role of p75NTR in regulating osteogenic differentiation is still controversy. Studies demonstrated that p75NTR‐positive adult human multipotent MSCs displayed increased osteogenic differentiation in vitro than p75NTR‐negative MSCs,^11^, ^35^ while Yoshikazu Mikami et al reported that p75NTR inhibited differential mineralization in C3H10T1/2 cells in a Trk‐independent manner.^17^ Furthermore, p75NTR was found to be an early surface marker during the first phase of jaw periosteum‐derived cell osteogenic differentiation in vitro.^36^ However, these findings were determined in postnatal models rather than progenitor cells, and the underlying mechanisms by which p75NTR regulates osteogenic differentiation in the initial maxillofacial developmental stage have rarely been investigated. Additionally, there are currently no research exploring the alveolar bone development on the p75NTR knockout mice. Here, we reported for the first time that deletion of p75NTR reduced alveolar bone mass and inhibited osteogenic differentiation ability in mice, supporting the findings of our in vitro studies on the effect of p75NTR in regulating osteogenic differentiation of EMSCs.

Given the reduced bone mass and osteogenic differentiation in p75NTR^−/−^ mice, we focused on embryonic stem cells to reveal the role of p75NTR in regulating craniomaxillofacial development. CNC‐derived EMSCs, the progenitor cells of craniofacial hard tissues including the maxilla and mandible, populate the majority of the first branchial arch mesenchyme and play a crucial role in tooth and mandibular morphogenesis.^20^ In previous studies, we successfully developed rat EMSCs and detected their multi‐differentiation abilities.^10^, ^37^, ^38^ In this study, EMSCs were isolated from WT and p75NTR^−/−^ embryos separately, but derived from the same heterozygous pregnant mice. Thus, hereditary differences between the two kinds of experimental EMSC populations were avoided, increasing their comparability. Detection of cell surface molecules suggested that both of these cell populations originated from CNCs. Moreover, we confirmed that p75NTR expression rate in wild‐type murine E12.5d EMSCs was 23.75%.

p75NTR has been reported to regulate osteogenic differentiation in several kinds of cells through different signalling pathway in vitro. However, the underlying mechanism of p75NTR in regulating osteogenic differentiation of murine EMSCs is still inconclusive. In this study, we found p75NTR positively regulated osteogenic differentiation ability of murine EMSCs. To clarify the regulatory mechanism of p75NTR, we performed RNA‐Seq of WT and p75NTR^−/−^ EMSCs after mineralized induction. KEGG pathway analysis showed that the differentially expressed genes were highly involved in the PI3K/Akt signalling pathway. Interestingly, there are increasing evidence supporting that the PI3K/Akt pathway is required for murine cell osteogenesis and bone tissue metabolism.^39^, ^40^, ^41^, ^42^, ^43^, ^44^ It has been reported that Runx2 enhances PI3K/Akt signalling *via* upregulating the expression of PI3K subunits and Akt, while the DNA binding of Runx2 and Runx2‐dependent transcription was also greatly enhanced by PI3K/Akt signalling in MC3T3‐E1 cells.^27^ This positive feedback loop indicates that Runx2 and PI3K/Akt signalling depend on each other to regulate osteoblast differentiation and that the PI3K/Akt pathway is important for the expression and activity of Runx2. In our study, PI3K/Akt signalling was required for murine EMSC osteogenic differentiation. Moreover, increased p75NTR was reported to activate the phosphorylation of PI3K and the Akt survival pathway in neuronal cultures.^45^, ^46^ It was reported that p75NTR increased Akt activation through a Trk‐independent pathway that requires PI3K.^45^ The effect of p75NTR on Akt correlates with increased tyrosine phosphorylation of the p85 regulatory subunit of PI3K and of Shc adaptor proteins. Furthermore, reports showed that LY294002, the inhibitor of PI3K, suppressed BMP2‐activated osteoblast differentiation.^39^, ^47^ Here, our experiments suggested that regulation of p75NTR influenced PI3K/Akt pathway activation during EMSC osteogenic differentiation. Additionally, suppression of PI3K/Akt signalling in p75NTR overexpressed EMSCs resulted in significantly downregulated osteogenic differentiation, while activation of PI3K/Akt pathway in p75NTR knock‐down EMSCs significantly upregulated the osteogenic differentiation. All these data suggested that the PI3K/Akt pathway, which is required for EMSC osteogenic differentiation, is targeted by p75NTR.

β‐catenin signalling is involved in bone formation. Higher levels of β‐catenin promoted bone formation with increased expression of osteoblast‐specific genes,^48^ while knock‐down of the β‐catenin gene caused abnormal osteoblast differentiation.^48^, ^49^ Studies showed that β‐catenin plays a direct role in osteoblast differentiation, and BMP2‐induced differentiation may be regulated by β‐catenin signalling.^50^, ^51^ The canonical Wnt pathway promotes bone formation through β‐catenin/TCF1‐mediated activation of the master osteogenic transcription factor Runx2.^52^ Moreover, it was previously demonstrated that the Wnt/β‐catenin signalling pathway was targeted by p75NTR during osteogenic differentiation in rat EMSCs.^34^ Consistent to this study, we found p75NTR regulated β‐catenin expression and osteogenic differentiation in murine EMSCs in our experiment. During the osteogenic differentiation of human umbilical cord mesenchymal stem cells, activated PI3K/Akt signalling promoted the phosphorylation of GSK‐3β, leading to the high concentration accumulation and stabilization of β‐catenin to activate the transcription of Runx2.^30^ Furthermore, activation and accumulation of β‐catenin regulated by Akt were reported to play an important role in cell osteogenic differentiation.^53^, ^54^, ^55^, ^56^ In this study, regulation of PI3K/Akt signalling caused by p75NTR also influenced activation of β‐catenin, which was possibly involved in osteogenic differentiation in EMSCs. Accordingly, we speculate that p75NTR promotes osteogenic differentiation of the murine EMSCs partly through PI3K/Akt/β‐catenin signalling.

During the healing of bone fracture, increased expression of NGF was detected^57^ and NGF promoted the osseointegration and regeneration of inferior alveolar nerve.^58^ Consistent with this, our results showed that the production level of NGF in murine EMSCs was gradually increased during osteogenic differentiation. These results indicated that NGF are possibly involved in osteogenic differentiation *via* autocrine and paracrine pathways. P75NTR, a single membrane‐spanning protein, bound to NGF with its extracellular domain.^59^ Here, the mutant mice we used were disrupted in the extracellular domain of p75NTR, lacking a functional p75NTR.^31^ It was demonstrated that pro‐BDNF inhibited the proliferation and migration of OLN‐93 oligodendrocytes through the p75NTR^ECD^ signal pathway.^60^ However, protective activity of p75NTR in regulating cell survival is neither extracellular ligand‐dependent nor receptor‐dependent.^61^ Therefore, we determined to explore whether the p75NTR^ECD^ is essential for NGF‐regulated osteogenic differentiation. We added exogenous NGF to osteogenic differentiation medium and found enhanced osteogenic differentiation in WT rather than p75NTR^−/−^ EMSCs, suggesting p75NTR plays a crucial role in NGF‐mediated osteogenic differentiation, and p75NTR^ECD^ is important for osteogenic differentiation of murine EMSCs. Furthermore, the PI3K/Akt/β‐catenin signalling was also enhanced by NGF in WT rather than p75NTR^−/−^ EMSCs, indicating that p75NTR^ECD^ may plays an important role in facilitating NGF‐induced PI3K/Akt/β‐catenin signalling activation during osteogenic differentiation.

In conclusion, our experiments demonstrated that the deletion of p75NTR reduced alveolar bone mass and may attenuated the alveolar bone development in mice. P75NTR positively regulated osteogenic differentiation of EMSCs *via* enhancing the PI3K/Akt/β‐catenin pathway. Moreover, p75NTR^ECD^ seemed to play an important role in NGF promoted osteogenic differentiation in murine EMSCs. Given that EMSCs are the progenitor cells of craniofacial hard tissues including the maxilla and mandible, it is important to investigate the mechanism of EMSC osteogenic differentiation. Our findings may enrich the understanding of maxillofacial development and support the use of p75NTR and PI3K/Akt/β‐catenin pathway as potential targets for management of alveolar bone engineering.

## CONFLICTS OF INTEREST

None.

## AUTHOR CONTRIBUTIONS

All authors participated in study design, data interpretation and analysis and manuscript review. Yingying Wang, Kun Yang and Gang Li performed the experiences. Rui Liu and Junyu Liu contributed to data analyses. Jun Li and Mengying Tang performed the data analyses and edited the manuscript. Yingying Wang prepared all figures and wrote the main manuscript. Manzhu Zhao, Jinlin Song and Xiujie Wen designed the present study.

## Supporting information

Table S1

## Data Availability

The data that support the findings of this study are available from the corresponding author upon reasonable request.
