# Supplementary material for: p75NTR−/− mice exhibit an alveolar bone loss phenotype and inhibited PI3K/Akt/β‐catenin pathway
Source: Cell Prolif. 2020 Mar 25;53(4):e12800. doi: 10.1111/cpr.12800 (PMC7162804; doi:10.1111/cpr.12800)
Supplement: Supplementary file 1 — Table S1 [file CPR-53-e12800-s001.docx]

**Supplemental Table 1 Primer sequences**

| Gene | Primer sequence | GenBank accession no. |
| --- | --- | --- |
| P75NTR | Forward: 5′- AGCCCTCAAGGGTGATGGC-3′  Reverse: 5′- CCTCGTGGGTAAAGGAGTCTATATG-3′ | NM_033217 |
| Runx2 | Forward: 5′- GACTGTGGTTACCGTCATGGC-3′  Reverse: 5′- ACTTGGTTTTTCATAACAGCGGA-3′ | NM_001146038 |
| Col1 | Forward: 5′- AAGTCACCGAGAGAATTGTCAC-3′  Reverse: 5′- AGAGAGCCTGTCTTAGCATATCC-3′ | NM_007732 |
| β-catenin | Forward: 5′- ATGGAGCCGGACAGAAAAGC-3′  Reverse: 5′- TGGGAGGTGTCAACATCTTCTT-3′ | NM_001165902 |
| GAPDH | Forward: 5′- GGCTGTATTCCCCTCCATCG-3′  Reverse: 5′- CCAGTTGGTAACAATGCCATGT-3′ | NM_008084 |
